# New strategies for managing adult gliomas

**DOI:** 10.1007/s00415-020-09884-3

**Published:** 2020-06-15

**Authors:** Alastair J. Kirby, Gerald T. Finnerty

**Affiliations:** 1 Department of Basic and Clinical Neuroscience, King’s College London, De Crespigny Park, London, SE5 8AF UK; 2 Department of Neurology, King’s College Hospital NHS Foundation Trust, Denmark Hill, London, SE5 9RS UK

**Keywords:** Glioblastoma, Cancer, Seizure, Treatment resistance, Tumour recurrence, Malignant progression

## Abstract

Gliomas are hard to treat. Their prognosis has improved little over the past few decades. Fundamental therapeutic challenges such as treatment resistance, malignant progression, and tumour recurrence persist. New strategies are needed to advance the management and treatment of gliomas. Here, we focus on where those new strategies could emerge. We consider how recent advances in our understanding of the biology of adult gliomas are informing new approaches to their treatment.

## Introduction

Gliomas are the most frequent primary brain tumour in adults [1]. Unlike many other cancers, the prognosis for gliomas has improved little over recent decades [2]. The standard of care is maximal surgical resection with adjuvant chemotherapy and radiotherapy [3–7]. Disappointingly, the 1- and 5-year relative survival rates for patients of all ages with glioblastoma are 41% and 7%, respectively [8]. The age at presentation combined with their poor prognosis means that people with gliomas suffer the greatest loss of “years of potential life” for any adult cancer [9].

Two features of gliomas mean that they are particularly hard to treat and make them prone to recurrence. First, the majority of gliomas are highly invasive [10]. Consequently, the glioma has no clear boundary, which makes it very hard for surgeons to remove the entire tumour. Even removing an entire cerebral hemisphere may not cure [11]. Second, a glioma is not composed of a single clone of cells. Instead, each glioma contains multiple cell types at varying developmental stages and with different genetic and epigenetic signatures [12–14]. Hence, gliomas do not present a well-defined target for (neo)adjuvant chemoradiotherapy.

In this review, we focus on emerging strategies to manage and treat adult gliomas. The 2016 WHO classification of brain tumours has transformed brain tumour diagnosis by combining molecular and histological features of gliomas. Since then, there have been several excellent recent reviews on different types of gliomas [15–17], standard of care for gliomas with management guidelines [6, 7], and treatment biomarkers [16]. Here, we consider how recent advances in our understanding of the biology of gliomas are informing new and different clinical approaches to tackle treatment resistance, tumour recurrence, and malignant progression.

## Genesis of gliomas

Advances in our understanding of gliomagenesis have led to new strategies for treating gliomas. Two influential ideas have been clonal evolution and the glioma stem cell hypothesis, which is an example of the more general cancer stem cell hypothesis.

Clonal evolution is thought to be a feature of many cancers [18, 19]. Essentially, tumour cells have changes in their DNA, which enable them to replicate without regulation. The clones of tumour cells that are well adapted to their environment proliferate. The emergence of multiple clones of tumour cells and clonal selection can explain the heterogeneity of cell types in tumours, treatment resistance, and tumour recurrence.

The glioma stem cell hypothesis is a more recent idea. Glioma stem cells are a small, sub-population of the glioma cells, which are able to proliferate, self-renew, and propagate the entire tumour when transplanted [20]. They have been isolated from human glioblastomas [20–22]. The signalling pathways in glioma stem cells are modified to suppress apoptosis and enhance DNA repair, which fosters tumour growth [23]. The progeny of the glioma stem cells develop to varying extents, which generates the phenotypic variability of the glioma cells that form the bulk of glioblastomas [21]. It has been harder to show that glioma stem cells are present in less malignant adult gliomas. Evidence for their existence has come from single-cell RNA sequencing of lower grade gliomas, but it has not yet been shown that glioma cells with stem-like RNA signatures can generate an entire tumour when transplanted [24, 25].

Clonal variation and the cancer stem cell hypothesis were initially thought to be distinct explanations for tumour heterogeneity [26], but others have argued that they should be integrated [27, 28]. The combined hypothesis approach has been applied to gliomagenesis. A first step is that clonal evolution selects glioma cells that have some of the properties of stem cells. Taking this further, glioma stem cells have been proposed to be an end product of clonal variation [29]. The progeny of the glioma stem cells take different differentiation pathways, which contributes to glioma cell heterogeneity [25].

## Clinical significance: gliomagenesis and therapies

Knowledge of the genetic mutations and the resulting tumour proteins implicated in gliomagenesis has opened avenues for the development of new precision medicine treatments, some of which are undergoing trials. For example, mutations in the isocitrate dehydrogenase 1 (IDH1) gene have been identified as driver mutations in many gliomas [30, 31]. Small molecule inhibitors of the mutant IDH1 protein have been developed to target glioma cells [32].

Immunotherapies have taken advantage of our molecular understanding of gliomas too. Gliomas present fragments of tumour-specific proteins called neoantigens on their cell surface. Peptide vaccines have been developed to prime the immune system to attack cells presenting tumour neoantigens. Neoantigens derived from mutant IDH1 were an early target in preclinical models [33]. More recent peptide vaccines trialled in humans target multiple surface proteins specific to the patient’s glioma to boost the immune response [34, 35]. These vaccines have generated promising results in early studies, but have yet to be validated in large clinical trials. Similarly, in another immunotherapy, chimeric antigen receptor (CAR) T cells have been modified to express a molecule, chlorotoxin, that binds specifically to glioblastoma cells and boosts the treatment response [36].

The glioma stem cell hypothesis suggests further approaches to treating gliomas. It implies that glioma growth would stop if all the glioma stem cells were killed [26]. The therapeutic problem is that glioma stem cells are resistant to chemotherapy [37] and radiotherapy [23]. Attention has turned to the microenvironment involving the glioma and surrounding brain. Gliomas hijack the specialised intercellular signalling that occurs between healthy cells in brain spaces termed niches to facilitate glioma survival and growth. This is particularly true for the perivascular space termed the vascular niche. The perivascular space is a major route used by glioma stem cells to invade the brain [38]. Glioma stem cells are attracted to blood vessels by molecules, such as bradykinin, released by the endothelial cells [39], and induce angiogenesis to promote tumour growth [40, 41]. This suggests that inhibiting the signalling between endothelial cells and glioma stem cells in the vascular niche would be therapeutically advantageous. Great attention has been focused on vascular endothelial growth factor A (VEGF-A), which is released by glioma cells and promotes angiogenesis. However, monoclonal antibodies to VEGF-A, which inhibit angiogenesis, have failed to improve overall survival of patients with newly diagnosed glioblastoma [42, 43] or progressive glioblastomas [44]. As a result, other signalling pathways in the tumour microenvironment are being investigated as therapeutic targets. For example, the EphA3 tyrosine kinase receptor is highly expressed on glioblastoma cells with stem-like properties, but is present in low levels in healthy adult tissue [45]. A clinical trial (NCT03374943) of monoclonal antibodies against EphA3 in glioblastoma patients has started.

## Glioma cellular heterogeneity

The ability to study the molecular profiles of large numbers of single cells enables heterogeneity within gliomas to be probed in detail. Primary glioblastomas (WHO grade IV) are highly aggressive tumours. Molecular profiling using genomic, epigenomic, and single-cell transcriptomic information indicates that individual glioblastoma cells converge on one of four main cellular states: astrocyte-like, mesenchymal-like, oligodendrocyte-like, and neural progenitor-like [46]. Each glioblastoma contains cells from all four states, with one state predominating. Notably, glioblastoma cells can change their molecular profiles and move between states [46]. Hence, killing all the glioblastoma cells in one cellular state will have limited benefit, because the remaining glioblastoma cells will repopulate the “lost” state when treatment ceases. Instead, effective treatment requires that all states are targeted.

## Clinical significance: cellular heterogeneity

Glioma cell heterogeneity has led to a re-evaluation of treatment biomarkers. Temozolomide is an alkylating agent that is the backbone of chemotherapy treatment for gliomas. The effect of Temozolomide is reversed by the endogenous enzyme, *O*^6^-methylguanine DNA methyltransferase (MGMT). The methylation status of the *MGMT* gene promoter controls MGMT protein expression and, therefore, the response of gliomas to alkylating chemotherapy agents [47]. The methylation status of the *MGMT* promoter is measured to determine whether Temozolomide should be given. However, the methylation status of single cells varies within individual gliomas [48]. Importantly, alkylating chemotherapy agents can benefit gliomas with “borderline” methylation status [49]. These findings suggest that multiple biopsies from a glioma are required to fully characterise its methylation status and to guide treatment [50]. This proposal needs to be tested in a clinical trial to see whether patient outcomes are affected.

## Glioma: brain interaction modifies the tumour microenvironment

Glioma cells change their cellular state and molecular profile in response to the microenvironment within and around the tumour [46]. The glioma microenvironment is not just created by ions and molecules released by glioma cells. The brain contributes too by adapting to the glioma growing within it, for example, by activating neuronal plasticity mechanisms. Hence, the glioma environment is a function of both the glioma and the surrounding brain.

The neocortex around gliomas becomes hyperexcitable leading to seizures [51, 52]. It has been proposed that neuronal activity increases the proliferation of high-grade gliomas [53, 54]. Experiments in mouse glioma models suggest that the mechanism is based on the release of the ectodomain of a postsynaptic cell-adhesion molecule, neuroligin-3 (Nlgn3), which acts as a growth factor. Synaptic Nlgn3 is cleaved in response to synaptic activity. The extracellular domain is shed into the extracellular space and diffuses to the glioma cells where it activates glioma signalling pathways that result in cellular proliferation [53, 54]. Blocking the enzyme, ADAM10 that cleaves synaptic Nlgn3 is one therapeutic approach to disrupt this mechanism for glioma growth.

The idea of a direct interaction between a glioma and the surrounding brain has developed further with the finding that neurons form putative synapses on glioma cells. Ultrastructural analysis of human glioma cells transplanted into mouse brains revealed that the glioma cells form structures with neighbouring axons [55–57]. These structures are similar to the excitatory glutamatergic synapses formed between neurons. Glioma cells express a variety of synaptic transmitter receptors [58–60], which enable paracrine signalling to glioma cells. The existence of functional neuron-to-glioma communication was tested by activating the peritumoural cortex. The glioma cells exhibited a rapid depolarisation with a prominent AMPA-receptor-mediated component, recapitulating excitatory neural transmission [55]. Inhibition of AMPA receptors in xenograft models increased overall survival of the mice by reducing migration and proliferation [55, 56]. In a proportion of glioma cells, the depolarising current spread through gap junctions between glioma cells. The depolarization wave is accompanied by a transient increase in calcium concentration within the glioma cells, which promotes glioma cell invasiveness [55]. These findings suggest that glioma cells integrate themselves into neural circuits. The consequence is that neuronal activity may promote glioma proliferation and invasion.

The evidence above considered how neural circuitry in the brain affects gliomas. Newly emerging evidence suggests that the reverse happens; that is, gliomas modify neural circuitry. An early idea was that gliomas caused seizures, because glutamate release from glioma cells resulted in greater excitotoxic cell death of inhibitory interneurons than of excitatory pyramidal cells in the peritumoural cortex [51, 52, 61]. A more nuanced idea has emerged from a mouse glioblastoma model that incorporates mutations in the RTK–RAS–PI3K pathway. Two mutations in the catalytic subunit of the PI3K enzyme, PI3KCA, were identified that altered the expression of synapse-associated genes [62]. Mice with these two mutations developed seizures due to reduced inhibitory synapses and increased excitatory synaptic synapses between neurons in peritumoural cortex. One of the mutations increased the expression of glypican, which promotes the formation of excitatory synapses [63]. When glypican production in the glioma cells is blocked, the mice had fewer seizures and survived longer [62].

Collectively, the recent evidence suggests that a subset of gliomas forms a positive feedback loop with the surrounding peritumoural cortex. The therapeutic challenge now is to break this feedback loop in a way that does not disrupt neural activity and impair cognition.

## Clinical significance: glioma-associated seizures

Epileptic seizures are the most common presenting symptom in patients with glioma [64, 65]. This is concerning, because the recent experimental evidence suggests that aberrant neuronal activity in the surrounding peritumoural cortex facilitates glioma growth [53, 61]. Low-grade glioma patients that present with seizures have longer overall survival if their seizures cease [66]. Furthermore, effective seizure control is the best predictor of quality of life in glioma patients [67]. Consequently, effective seizure control is critically important for people for gliomas. However, glioma-associated seizures frequently respond poorly to anticonvulsant medication [68]. Surgical removal of the epileptic focus during glioma surgery controls seizures in some patients, but not all [69]. New anticonvulsants are needed.

## Malignant progression

Some gliomas have an indolent course initially and then behave more aggressively. The terminology around this has been re-evaluated. Malignant transformation was used to describe low-grade (WHO grade I–II) gliomas developing features of high-grade (WHO grade III–IV) tumours. The practise has been undermined by molecular profiling of gliomas, which gives a more accurate prognosis [15]. Increasingly, WHO grade II and grade III gliomas with the same molecular profile are grouped together, because they have a similar prognosis and are referred to as lower grade gliomas [15, 70, 71]. The change from indolent to more aggressive behaviour is better termed malignant progression.

We do not understand what causes malignant progression and where it occurs. Genetic changes associated with malignant progression have been identified [71, 72]. However, there has been less progress in understanding how those genetic changes underpin more aggressive glioma behaviour. In large part, this is due to the lack of suitable experimental models. True lower grade cell lines have been hard to obtain. Glioma stem cells have been isolated from adult higher grade gliomas, but are challenging to isolate from adult lower grade gliomas. As a result, standard in vitro or xenograft models have been challenging to set up [32, 73, 74]. New model systems are needed.

## Clinical significance: malignant progression

**Fig. 1:**
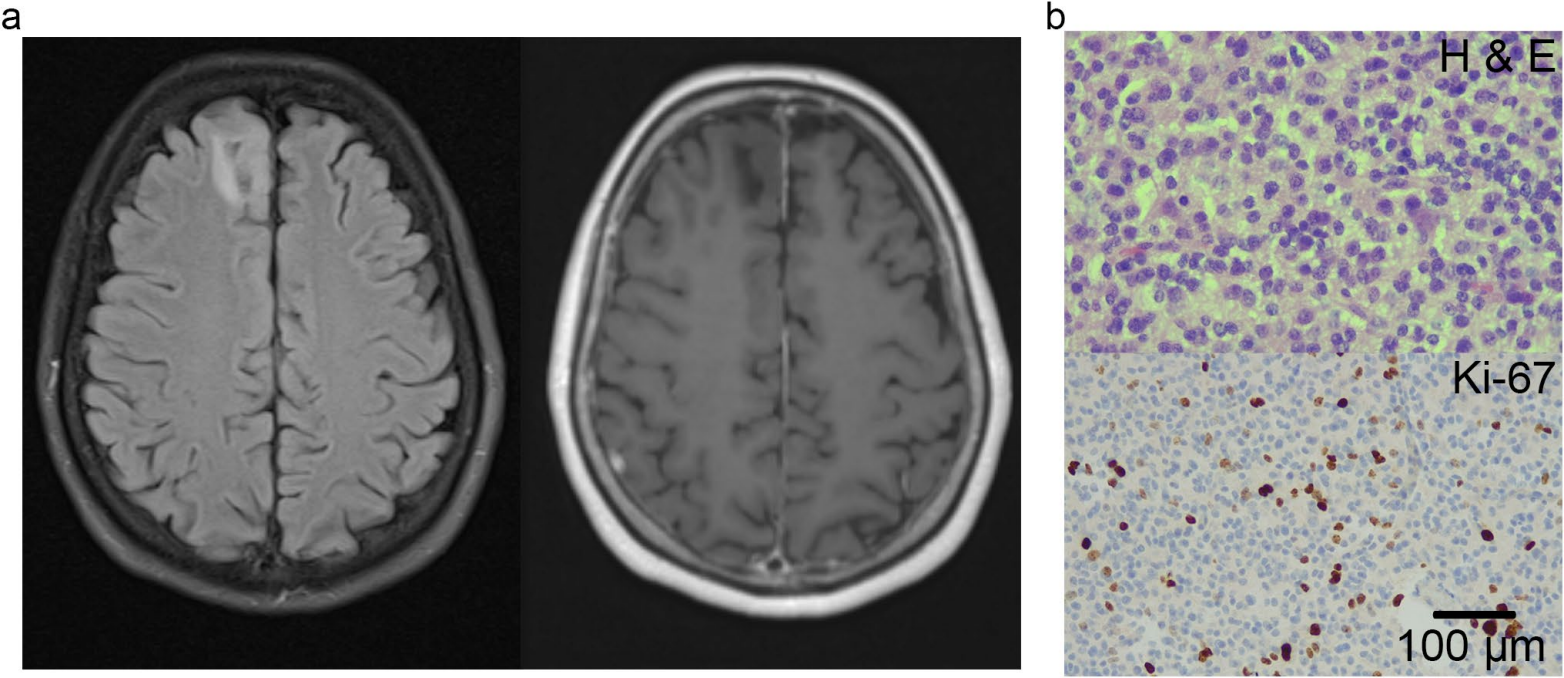
Predicting malignant transformation in low-grade gliomas. **a** Patient presented with a generalised seizure. Left panel, FLAIR MRI, lesion in the right superior frontal gyrus. Right panel, T1-weighted MRI, lesion showed no contrast enhancement. No increase in perfusion (data not shown). Radiological diagnosis, low-grade glioma. **b** Neuropathological diagnosis: IDH2 mutant, 1p/19q co-deleted, anaplastic oligodendroglioma (WHO grade III). Top panel, H&E stain exhibiting mitotic figures and apoptotic nuclei, no necrosis, or microvascular proliferation. Bottom panel, Ki-67-positive nuclei stain brown. Proliferation rate ~ 10%. Scale bar; 100 µm

Malignant progression is a key clinical issue in the management of gliomas, particularly as lower grade gliomas commonly affect young adults. Malignant progression is frequently detected neuroradiologically by contrast enhancement or increased perfusion on MRI scans. However, these findings are not always present (Fig. 1). The issue is that the neuroradiological measures focus on vascular changes and not on the glioma cells. Consequently, they detect malignant progression when it is established rather than at the earliest stages. New biomarkers to predict early stage malignant progression are needed.

## Liquid biopsy

Tissue biopsies give a snapshot of a glioma. However, the molecular profile of glioma cells is not static. It evolves. Ideally, treatment would change to mirror progression of the glioma. A minimally invasive biomarker based on the molecular profile of glioma cells could provide valuable information about tumour evolution. Such a biomarker could also be used to diagnose chemoradiotherapy-induced changes in brain imaging termed pseudoprogression and to monitor response of the glioma to treatment.

There has been huge interest in studying body fluids, such as blood or cerebrospinal fluid, to detect evidence of cancer. This is referred to as liquid biopsy. Blood biomarkers include circulating tumour cells, extracellular vesicles, and cell-free tumour DNA (ctDNA) [75]. Low frequencies of both circulating tumour cells and extracellular vesicles have been identified in the blood of glioma patients. The detection of circulating tumour cells in the blood of glioma patients is highly variable due to the use of different isolation techniques [75]. Extracellular vesicles have been shown to be significantly higher in gliomas with true progression than in patients with pseudoprogression or stable disease [76].

Blood biomarkers have the huge advantage of ease of collection. However, the blood–brain barrier limits access of tumour tissue to the blood. Tests of blood-derived extracellular vesicles and circulating tumour cells have suffered with low sensitivity [77]. As a result, they have not yet reached the clinic.

**Fig. 2:**
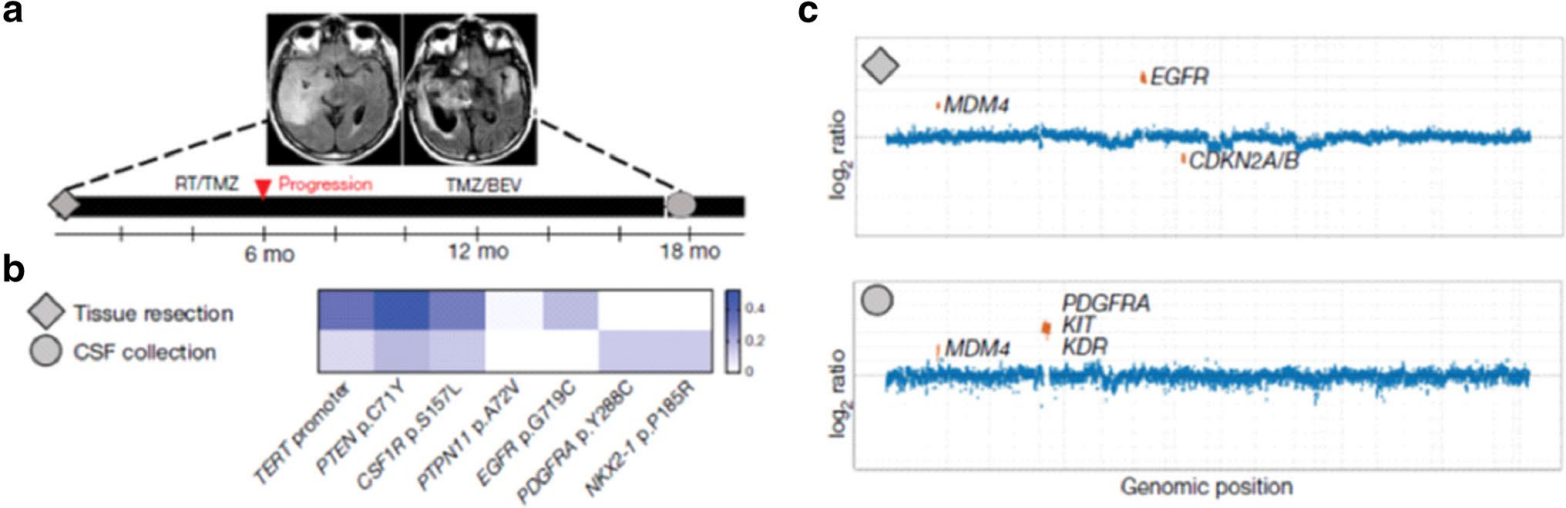
Cell-free DNA analysis of CSF to follow tumour evolution. **a** Fluid attenuated inversion recovery MRI showing glioblastoma at the time of resection (grey diamond) and time of CSF collection (grey circle). **b** CSF showed a new platelet-derived growth factor receptor alpha (PDGFRA) mutation. **c** Copy-number variation plots. CSF showed PDGFRA gene amplification and loss of the epidermal growth factor receptor (EGFR) gene amplification found in the original sample. Figure adapted from Miller et al. Nature 2019 [79]

Liquid biopsy based on cerebrospinal fluid offers an approach that circumvents the blood–brain barrier. Analysis of ctDNA in the CSF successfully identified glioma in approximately half of patients [78, 79]. The probability of identifying ctDNA in the CSF was more successful in higher grade gliomas, suggesting that the presence of ctDNA in CSF could be used to study tumour progression (Fig. 2) [79].

Liquid biomarkers offer a minimally invasive alternative to tissue biopsies. Currently, blood- and CSF-derived biomarkers lack diagnostic specificity, due to the short half-life, penetrance, and dilution of the biomarker [75]. These limitations may be overcome with interventions such as transcranial-focused ultrasound that enhances the release of the biomarkers into the blood or CSF [80].

## Clinical significance: liquid biopsy to diagnose glioma mimics?

**Fig. 3:**
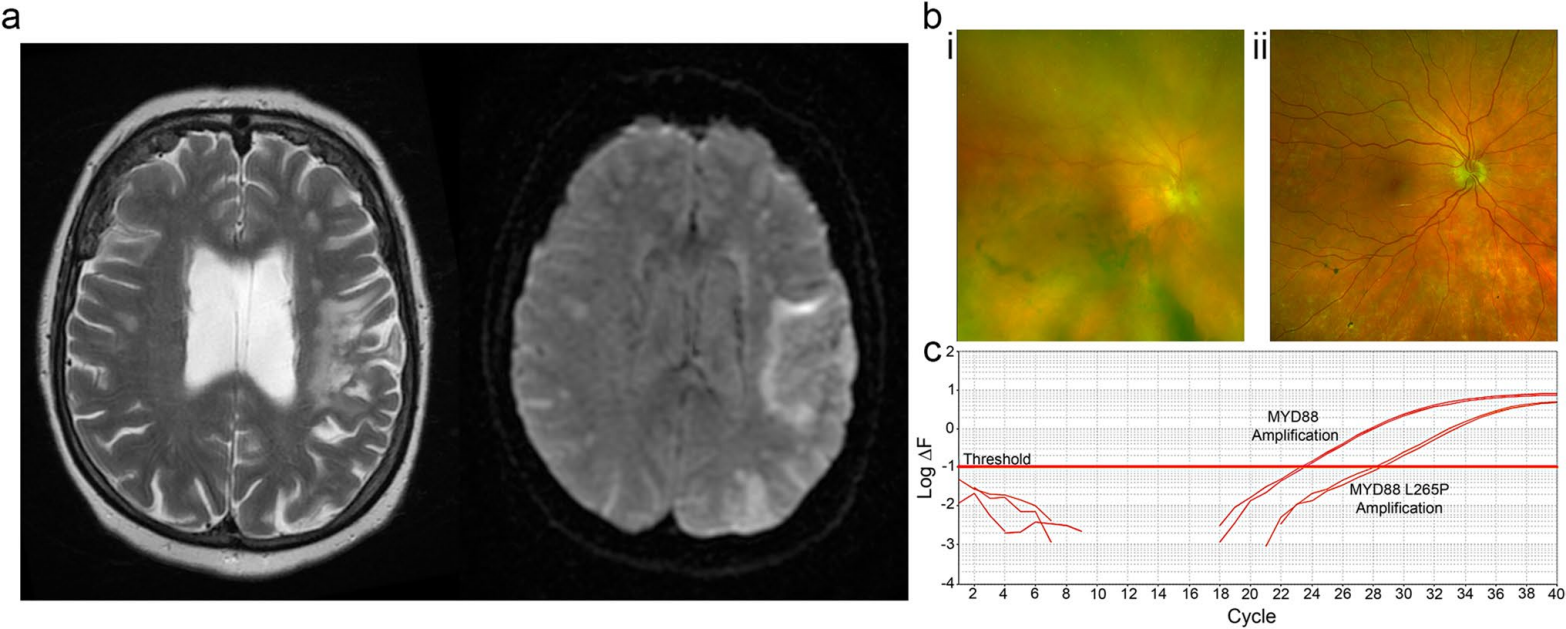
Diagnosis of primary CNS lymphoma with vitreous biopsy. **a** Patient presented with right-sided weakness with stuttering progression over several months. Left panel, T2-weighted MRI, increased signal in the territory of the left middle cerebral artery. Right panel, Diffusion-weighted imaging, increased signal suggesting restricted diffusion in left-hemisphere white matter. **b** Photographs of right fundus for patient in **a**: (i) before vitreous biopsy, retina obscured by cloudy vitreous; (ii) post-vitreous biopsy, retina visible, no sub-retinal deposits identified. **c** Detection of MYD88 L265P mutation by allele-specific PCR. The test compares the patient’s DNA with a positive control that has MYD88 L265P at 0.625%. The test relies on a failure of extension of the primers when there is mismatch between the primer and the extracted DNA. Any difference between the patient’s sample and the positive control is amplified by repeated PCR cycles and is measured (Δ*F*). The presence of the MYD88 L265P mutation is reported when the difference crosses a threshold

Liquid biopsy could be used to diagnose glioma mimics. A common diagnostic problem for neuro-oncology services is to differentiate glioblastoma from primary CNS lymphoma. Current practise is that tumour tissue is required from both glioblastoma and primary CNS lymphoma to diagnose the condition and to plan treatment [81]. Acquiring the tumour tissue is almost invariably done with a brain biopsy. Liquid biopsy may soon play a role here. The diagnosis of brain tumours is increasingly based on molecular features [82]. Glioblastoma and primary CNS lymphoma have different mutational landscapes [83, 84]. Therefore, it should be possible to differentiate the two conditions using liquid biopsy. This can be done with a vitreous biopsy as primary CNS lymphoma can involve the eyes (Fig. 3). Analysis of cell-free DNA in the cerebrospinal fluid has great potential, but has not yet reached the clinic.

## Translating basic science findings into glioma management

There has been considerable progress in our understanding of the biology of gliomas. However, many outstanding questions remain (Box 1). First, the finding that glioma growth and invasion is facilitated by aberrant neuronal activity suggests that effective control of seizures is vital for glioma patients. Novel agents to diminish neuronal hyperexcitability will need careful titration to avoid inhibiting neuronal plasticity, as this could impair cognitive function. Enzyme-inducing anticonvulsants need to be avoided as they can increase metabolism of some chemotherapy agents. Blood and CSF liquid biopsy have great potential as methods to diagnose glioma mimics and tumour pseudoprogression, and to monitor tumour progression. However, new approaches are needed to increase the sensitivity of liquid biopsies. What joins all the outstanding questions is the overwhelming need to translate the new knowledge about gliomas into novel strategies for their diagnosis and management.

## Box 1: Outstanding questions

1. Does control of seizure activity limit glioma growth and invasion?
2. Is killing glioma stem cells sufficient to reduce glioma growth or must all of the glioma cellular states be attacked?
3. Can new investigations predict which lower grade gliomas will progress into more malignant tumours?
4. Can the sensitivity and specificity of liquid biopsy be improved through interventions that increase release of glioma tissue fragments into the blood or CSF?
